# Genome-wide analysis of constitutional DNA methylation in familial melanoma

**DOI:** 10.1186/s13148-020-00831-7

**Published:** 2020-03-06

**Authors:** Catarina Salgado, Nelleke Gruis, BIOS Consortium, Marc Jan Bonder, René Luijk, Dasha V. Zhernakova, Matthijs Moed, Patrick Deelen, Martijn Vermaat, Maarten van Iterson, Freerk van Dijk, Michiel van Galen, Jan Bot, P. Mila Jhamai, Michael Verbiest, H. Eka D. Suchiman, Marijn Verkerk, Ruud van der Breggen, Jeroen van Rooij, Nico Lakenberg, Wibowo Arindrarto, Szymon M. Kielbasa, Peter van ’t Hof, Irene Nooren, Marian Beekman, Joris Deelen, Diana van Heemst, Alexandra Zhernakova, Ettje F. Tigchelaar, Morris A. Swertz, Bert A. Hofman, André G. Uitterlinden, René Pool, Jenny van Dongen, Jouke J. Hottenga, Coen D. A. Stehouwer, Carla J. H. van der Kallen, Casper G. Schalkwijk, Leonard H. van den Berg, Erik W. van Zwet, Hailiang Mei, P. Eline Slagboom, Cisca Wijmenga, Jan H. Veldink, Marleen M. J. van Greevenbroek, Cornelia M. van Duijn, Dorret I. Boomsma, Aaron Isaacs, Rick Jansen, Joyce van Meurs, Peter A. C. ’t Hoen, Lude Franke, Bastiaan T. Heijmans, Jan Oosting, Remco van Doorn

**Affiliations:** 1 Department of Dermatology, Leiden University Medical Center, Leiden, PO Box 9600, 2300 RC Leiden, The Netherlands; 2 Molecular Epidemiology, Department of Biomedical Data Sciences, Leiden University Medical Center, Leiden, The Netherlands; 3 Department of Pathology, Leiden University Medical Center, Leiden, The Netherlands

**Keywords:** Epimutation, Loss of imprinting, DNA methylation, Familial melanoma

## Abstract

**Background:**

Heritable epigenetic alterations have been proposed as an explanation for familial clustering of melanoma. Here we performed genome-wide DNA methylation analysis on affected family members not carrying pathogenic variants in established melanoma susceptibility genes, compared with healthy volunteers.

**Results:**

All melanoma susceptibility genes showed the absence of epimutations in familial melanoma patients, and no loss of imprinting was detected. Unbiased genome-wide DNA methylation analysis revealed significantly different levels of methylation in single CpG sites. The methylation level differences were small and did not affect reported tumour predisposition genes.

**Conclusion:**

Our results provide no support for heritable epimutations as a cause of familial melanoma.

## Introduction

Cutaneous melanoma is an aggressive form of skin cancer with a propensity to metastasize, causing significant mortality and health care expenditure. Approximately 10% of patients diagnosed with melanoma have a positive family history for this malignancy. In familial or hereditary melanoma, multiple melanoma cases aggregate in several generations of a family, consistent with an autosomal dominant inheritance pattern. A subset of familial melanoma cases is caused by germline mutations in the established high penetrance melanoma predisposition genes *CDKN2A* or *CDK4*. Recently, pathogenic variants in the *BAP1*, *TERT*, *POT1*, *TERF2IP*, *ACD* and *MITF* genes have been identified as a cause of familial melanoma. Several candidate melanoma susceptibility genes including *POLE*, *GOLM1* and *EBF3* have been reported [1–3]. However, in more than half of affected families, the cause of melanoma predisposition remains to be resolved despite much research effort. For this reason, the attention has turned to different mechanisms of inheritance including heritable epigenetic alterations. Clarifying the genetic basis of familial melanoma is clinically relevant as it would allow for genetic testing, risk estimation and targeted clinical surveillance of patients at high risk of melanoma.

Epimutations have been defined as heritable changes in gene activity due to DNA modifications, not encompassing changes in the DNA sequence itself [4]. It has been postulated to constitute an alternative mechanism to genetic mutation for cancer predisposition and commonly refers to constitutional promoter CpG island hypermethylation in all somatic cells of an individual [5]. The best-described example in cancer is hereditary nonpolyposis colorectal cancer (HNPCC, Lynch syndrome) where cases not affected by inactivating mutations in DNA mismatch repair genes were found to be caused by heritable promoter hypermethylation of the *MLH1* gene [6–8]. Epimutations have been classified as primary, occurring in the absence of an underlying DNA sequence alteration, and secondary, when a genetic mutation triggers the occurrence of an epigenetic modification [9]. Secondary epimutations in the *MSH2* and *DAPK1* gene have been identified in HNPCC and familial chronic lymphocytic leukemia, respectively [10–12].

Genomic imprinting causes certain genes to be silenced by DNA and histone methylation in a parent of origin-specific manner, ensuring proper expression during development. Loss of imprinting is a distinct epigenetic mechanism of disease, associated with deregulated gene expression that can be implicated in cancer development [13]. The association between loss of imprinting at the *IGF2–H19* locus at chromosome 11p15.5 and predisposition to Wilms tumour is an example of this epigenetic mechanism [14].

In familial melanoma, we and others have shown the absence of epimutation of the *CDKN2A* gene, the major high penetrance melanoma susceptibility gene [15, 16]. A previous study analysed methylation of 14 cancer-related genes in blood DNA from melanoma-prone family members. This analysis revealed no constitutional promoter hypermethylation, but reduced methylation of the *TNFRSF10C* promoter [17].

In this study, we aim to identify heritable epigenetic alterations that might account for familial clustering of melanoma in families where no genetic variants in established or candidate melanoma susceptibility genes were found. To this end, a genome-wide methylation analysis of peripheral blood DNA from patients with familial melanoma was performed. We assessed promoter hypermethylation of recently identified melanoma susceptibility genes and loss of imprinting and performed an unbiased analysis of hypermethylated CpG sites and regions.

## Results and discussion

Patients from 5 Dutch families with at least 3 melanoma cases in different generations were selected for this study (Table 1, pedigrees in Supplementary Figure 1). The presence of pathogenic gene variants in all currently established and candidate high penetrance melanoma susceptibility genes was assessed in all included cases using whole-genome sequencing. No germline mutations were found in these genes. To examine DNA methylation, DNA from peripheral blood of 2 affected members of each family (*n* = 10) was subjected to 450K Illumina arrays interrogating over 450,000 CpG sites (namely 483,891 probes after quality control) covering 99% of human genes following bisulfite conversion [18]. For comparative analysis, we could make use of DNA methylation data obtained from peripheral blood samples of a reference group of 1000 healthy Dutch individuals included in the Biobank-based Integrative Omics Studies (BIOS) consortium analysed using similar 450K arrays (raw data available from the European Genome-phenome Archive (EGA) under accession EGAS00001001077). All samples were compared individually to the reference group, while taking multiple testing into account using Bonferroni correction.

**Table 1:** Clinical characteristics of patients/families involved in the genome-wide analysis

Family	Number of CMM affected members	Patient number	Degree of kinship	Age at melanoma diagnosis	Age at DNA collection
I	9	I_1	2nd	62	76
		I_2		34, 34, 46, 48^a^	53
II	5	II_1	3rd	41	52
		II_2		52	56
III	4	III_1	2nd	51, 57^a^	68
		III_2		28	33
IV	3	IV_1	3rd	34	56
		IV_2		35	43
V	4	V_1	1st	49	55
		V_2		30	25

^a^Multiple primary melanomas diagnosed

**Fig. 1:**
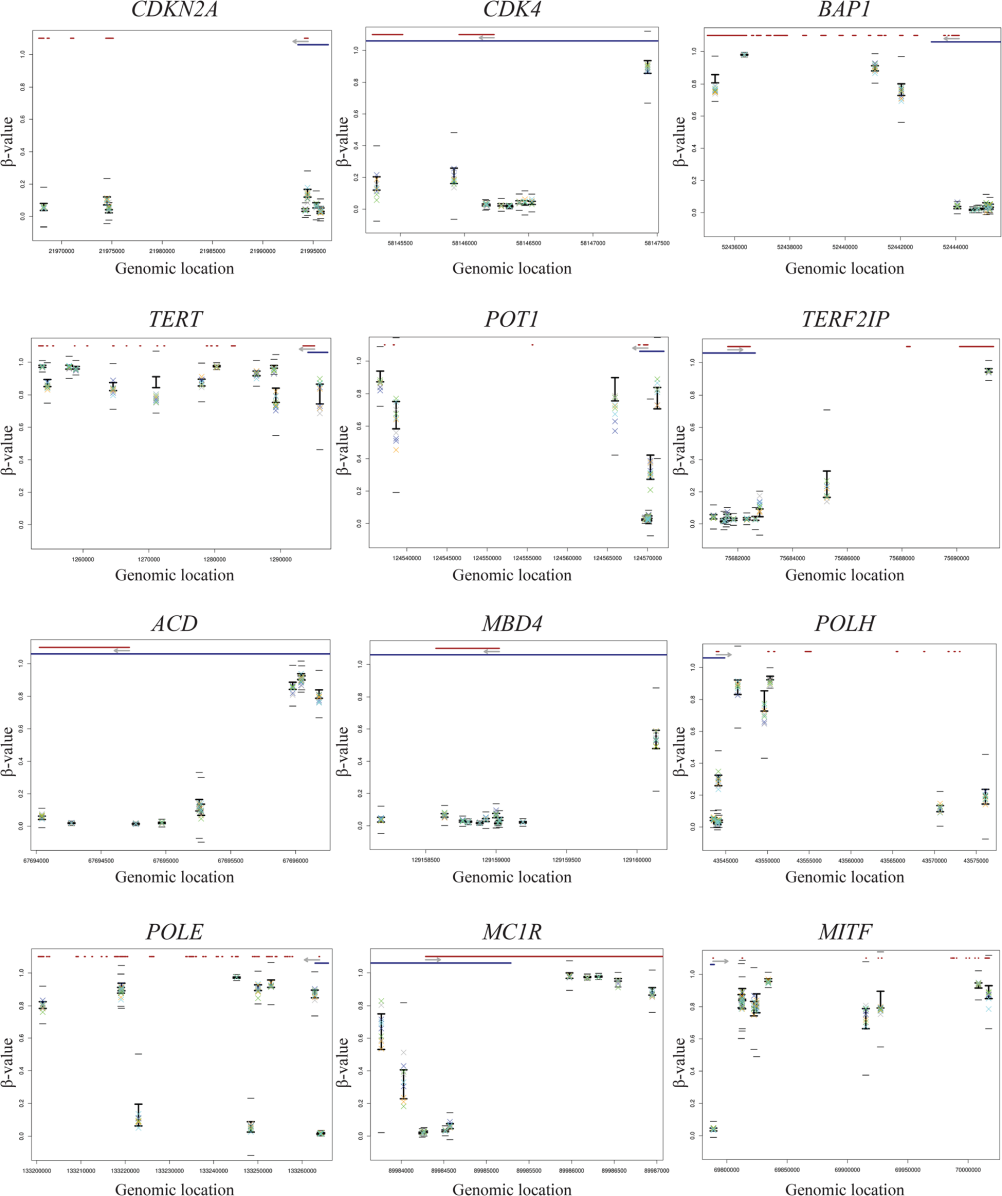
Methylation levels (*β* value) across the entire sequence of all established melanoma predisposition genes. In the upper part of each plot, the gene structure is represented in red and promoter region (“Promoter_associated” feature retrieved from Illumina annotation) in blue. The light grey arrow represents the transcription direction of the gene. For each CpG, the BIOS values are represented by the black vertical line with upper (average + 1 SD) and lower limits (average − 1SD). The families are represented as a X of different colours (family I—green, family II—blue, family III—yellow, family IV—light purple, family V—dark blue). To be considered as significantly different from the BIOS, the families’ symbols must go beyond the small black horizontal line (average ± 5.65 SD). Genes with more than 10 CpG sites assessed by 450K array were represented by 10 randomly selected CpGs. An extended version of figure 1 is available as supplementary information

First, we analysed the presence of promoter hypermethylation in the *CDKN2A*, *CDK4*, *BAP1*, *TERT*, *POT1*, *TERF2IP*, *ACD*, *MBD4*, *POLH*, *MITF*, *MC1R*, *POLE*, *EBF3* and *GOLM1* melanoma susceptibility genes. All CpG sites designated by a probe located across the entire sequence of these melanoma susceptibility genes in the familial melanoma samples were compared with reference samples. No significant difference (≥ *β* value average ± 5.65 SD) in methylation level, hypermethylation nor hypomethylation was found at any of these genes (Fig. 1).

The methylation status of imprinted genes in familial melanoma patients was then investigated. We checked all CpG sites in the entire gene sequence of all imprinted genes in humans (http://www.geneimprint.com/site/genes-by-species, accessed August 2019) interrogated by the arrays. The methylation levels of 4790 interrogated CpG sites located at these imprinted gene loci did not differ significantly (≥ *β* value average ± 5.65 SD) from the BIOS reference samples (Supplementary Figure 2). Since the regulation of imprinted genes is largely dependent on methylation levels, and there is no significant difference in any of the familial melanoma patients compared to BIOS, we conclude that there was no indication of loss of imprinting.

**Fig. 2:**
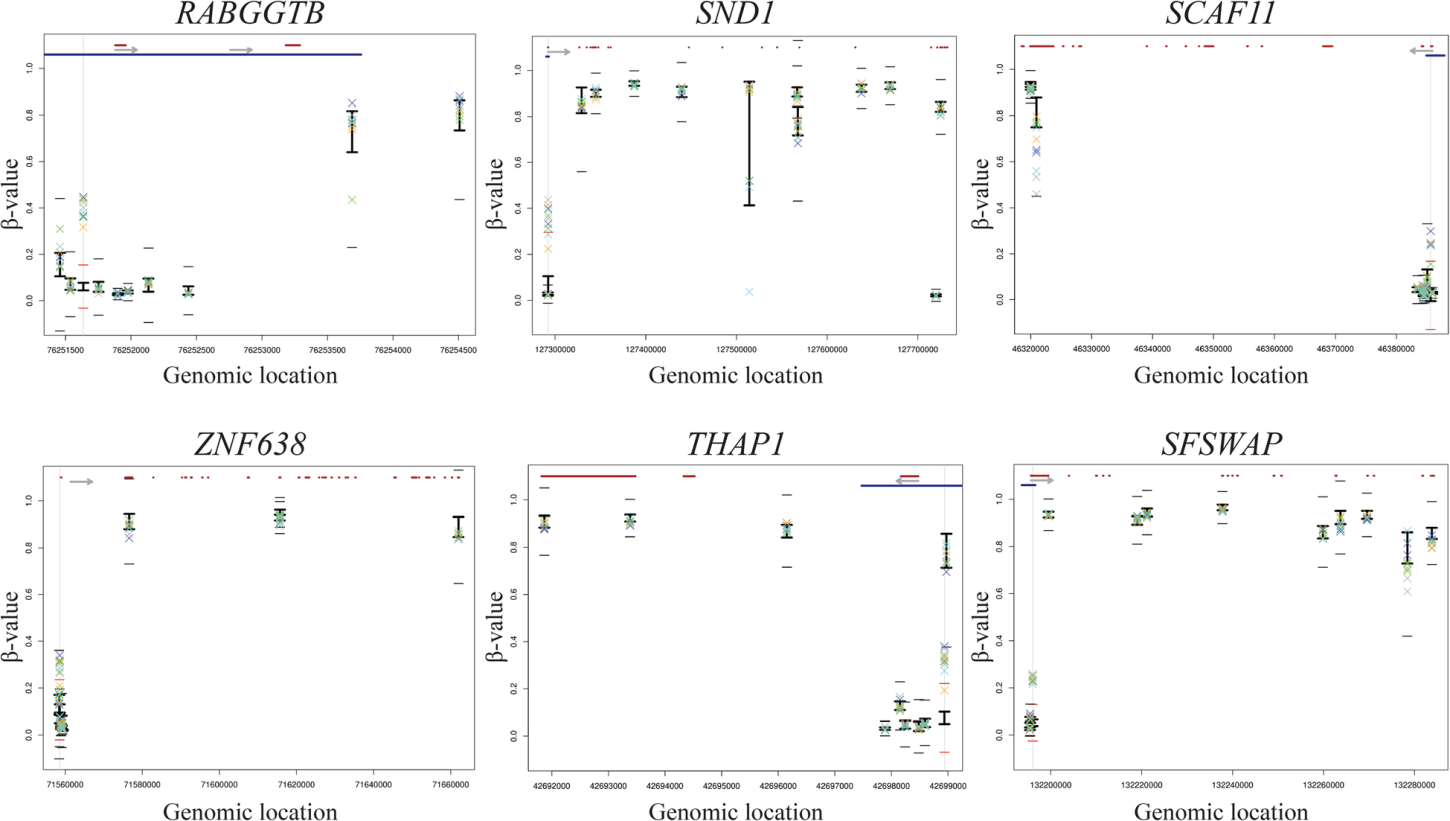
Methylation levels (*β* value) in all 6 significant upregulated CpGs located in the promoter regions of the genes. In the upper part of each plot, the gene structure is represented in red and promoter region (“Promoter_associated” feature retrieved from Illumina annotation) in blue. The light grey arrow represents the transcription direction of the gene. For each CpG, the BIOS values are represented by the black vertical line with upper (average + 1 SD) and lower limits (average − 1SD). The families are represented as a X of different colours (family I—green, family II—blue, family III—yellow, family IV—light purple, family V—dark blue). To be considered as significantly different from the BIOS, the families’ symbols must go beyond the small black horizontal line (average ± 5.65 SD). Genes with more than 10 CpG sites assessed by 450K array were represented by 10 randomly selected CpGs. The upregulated CpG in each plot is aligned with a vertical light grey line, and in this case, the little horizontal lines become red since the families’ symbols exceeded these limits. An extended version of figure 2 is available as supplementary information

Following analysis of candidate genes, we performed an agnostic genome-wide analysis by comparing DNA methylation of all interrogated CpG sites in the familial melanoma patients with those in healthy subjects. We considered as potential epimutations CpG sites located in gene promoters (using probes assigned to promoter regions according to annotation provided by Illumina) with significantly aberrant methylation levels in both members of an affected family compared to BIOS control samples. Since all reported cancer-predisposing epimutations were cases of constitutional promoter hypermethylation, we focused our analysis on this type of epigenetic event. All CpGs in promoters were assessed. Probes interrogating CpG sites lost due to single nucleotide variants, as identified using whole-genome sequence data, were not included in the analysis of the affected samples. We identified 6 single CpGs in gene promoters with significantly higher *β* values in both affected members of a melanoma family compared to healthy controls (Fig. 2, Table 2, Supplementary Table 1). In healthy controls, these CpG sites showed low average *β* values consistent with the absence of methylation. The CpG sites in the *RABGGTB*, *SND1*, *SCAF11*, *ZNF638*, *THAP1* and *SFSWAP* genes showed significantly higher Δ*β* values in both members of multiple families.

**Table 2:** Methylation levels (Δ*β* value) in all 6 significant upregulated CpGs in all subjects (*n* = 10)

CpG ID	Gene ID	BIOS control cases	Family I		Family II		Family III		Family IV		Family V	
			I_1	I_2	II_1	II_2	III_1	III_2	IV_1	IV_2	V_1	V_2
		*β* value^a^	Δ*β* value^b^									
cg21812670	RABGGTB	0.06	0.30	0.38	0.34	0.30	0.36	0.26	0.39	0.32	0.39	0.30
cg26642667	SND1	0.06	0.25	0.30	0.33	0.22	0.35	0.16	0.37	0.29	0.34	0.27
cg04385631	SCAF11	0.02	0.14	0.00	0.00	0.22	0.00	0.22	0.23	0.00	0.22	0.28
cg21843755	ZNF638	0.11	0.16	0.21	0.18	0.07	0.20	0.10	0.21	0.16	0.23	0.21
cg03301282	THAP1	0.08	0.26	0.23	0.20	0.27	0.25	0.12	0.29	0.27	0.30	0.24
cg02470959	SFSWAP	0.05	0.18	0.20	0.18	0.16	0.19	0.17	0.21	0.21	0.20	0.17

^a^Represents the average *β* value for the 1000 BIOS controls at each CpG site

^b^Represents the difference between BIOS average *β* value and patient *β* value at each CpG site

We assessed the methylation of contiguous interrogated CpG sites, and for all 6 cases, the hypermethylation was observed exclusively in the single identified CpG site, with neighbouring CpGs not showing significantly higher methylation levels. None of these genes have been reported as cancer predisposition genes by Rahman [19]. Only one of the CpGs is located in a cancer-related gene, *SND1*, according to Cancer Gene Census (http://cancer.sanger.ac.uk/census, accessed August 2019). This gene has no reported role in melanoma and functions as a gene fusion partner in certain malignancies. Given the established genetic heterogeneity, it is unlikely that the same epimutation would cause melanoma susceptibility in all 5 families. Together with the information about the function of the genes (Supplementary Table 1), we conclude that the identified hypermethylated CpG sites in these families do not appear to constitute plausible pathogenic high penetrance epimutation events.

Additionally, we evaluated CpG sites with significantly lower methylation levels in familial melanoma than in healthy control samples and found 35 hypomethylated CpGs in both members of a family (Supplementary Table 2). Fifteen CpG sites showed hypomethylation in all 5 families, suggestive of a batch effect as has been described for 450k methylation arrays [20]. Of the 35 hypomethylated CpG sites, only 2 were located in established cancer-related genes: *BRCA1*, an established breast and ovarian cancer susceptibility gene, and *ROS1*, encoding a receptor tyrosine kinase with a possible oncogenic role in melanoma [21]. For both genes, a single CpG site in the promoter demonstrated significantly lower methylation levels, with normal methylation of neighbouring CpG sites assessed by 450K array. For *BRCA1* and for *ROS1*, the CpG site was not part of a predicted transcription factor binding motif [22]. Hypomethylation of the *BRCA1* gene promoter has never been associated with transcriptional downregulation, and therefore, reduced methylation of this single CpG site in the *BRCA1* gene promoter is unlikely to have pathogenic significance. Expression of the *ROS1* oncogene is not known to be regulated by promoter methylation, but high expression has been associated with histone modifications and *EZH2* repression [23]. *β* values for the single CpG site in the distal promoter of the *ROS1* gene were 0.87 in control samples and approximately 0.65 in familial melanoma DNA samples. We consider it possible, but unlikely that lower methylation levels of a single CpG site in the distal promoter of *ROS1* would cause familial melanoma. Similar to the finding of *TNFRSF10C* hypomethylation in familial melanoma patients from the USA, which we could not detect in our patients, this finding might be analysed in a large number of melanoma families [17].

Since regions containing multiple CpG sites in promoters commonly work as units of transcriptional regulation, we additionally tried to identify differentially methylated regions. For this, we evaluated the average of all probes assigned to promoters for each gene comparing familial melanoma and healthy control samples. The annotation of the 450K array contains 13,715 genes with CpG probes assigned to promoters. The promoter of one gene (*CCNI*) showed significant higher methylation levels in 4 families, while promoters of the *CD47* and *USP46* genes had slightly higher methylation levels in 1 family each. Although statistically significant, the averaged promoter methylation level (*β* value) differences were minor, which does not support a relevant effect.

In this study, we analysed the possible occurrence of epimutations and loss of imprinting in familial melanoma using a genome-wide approach. A strength of the study is the selection of DNA samples from families with many affected members in multiple generations where no genetic cause could be identified and the availability of methylation data from a large cohort of 1000 Dutch healthy individuals for comparative analysis. There are some limitations to this study; the number of analysed families is small, and our results do not exclude the possibility that pathogenic epimutations might occur in a small proportion of melanoma families. Secondly, the 450K arrays interrogate CpG sites in almost all gene promoters, but do not cover all potentially regulatory sequences. In addition, we analysed blood DNA for the occurrence of epimutations, but certain epimutations might occur only in specific cell types in a mosaic state. In these patients with familial melanoma, we have not identified promoter hypermethylation of any melanoma predisposition gene, cancer predisposition gene or tumour suppressor gene. We have been able to determine several DNA methylation events that are candidate epimutations, methylation events shared by multiple members of a family that were not identified in healthy volunteers. However, it is not clear if the observed methylation alterations in these single CpG sites impact on expression of the respective genes. Based on the function of the genes and the fact that we did not identify a differentially methylated region, but only a single CpG site, we consider it is not plausible that any of the DNA methylation alterations that were detected constitute the cause of melanoma predisposition in these families. Moreover, given the established genetic heterogeneity, it is unlikely that the same epimutation would cause melanoma susceptibility in all 5 families. Therefore, we consider the observed CpG sites with higher and lower detected methylation levels to represent rare variations with no pathogenic significance or possibly the result of batch array effects. In summary, our results of genome-wide analysis provide little or no support for a role of heritable DNA methylation alterations as a cause of familial melanoma.

## Materials and methods

We selected 5 unrelated Dutch families with 3 or more melanoma cases in multiple generations and tested negative for germline mutations in the established high penetrance melanoma susceptibility genes *CDKN2A*, *CDK4*, *BAP1*, *TERT*, *POT1*, *TERF2IP*, *ACD* and *MITF* by next-generation sequencing (Fig. 1). Some patients had developed multiple melanomas. The majority of the melanomas were of the superficial spreading or nodular subtypes. The study was approved by the Leiden University Medical Center institutional ethical committee and was conducted according to the Declaration of Helsinki principles. DNA from 2 affected members from 5 families was isolated from whole blood samples. DNA was bisulfite-converted using the EZ DNA methylation kit (Zymo Research, D5001) and hybridized to Illumina 450K arrays (Illumina). The reference group encompassed 1000 whole blood DNA samples of healthy individuals included in the Biobank-based Integrative Omics Studies (BIOS) Consortium analysed with Illumina 450K arrays under similar conditions [24]. The median age of patients during blood sampling was 54 years, and for the 1000 healthy controls, it was 55 years. Sample quality control was performed using *MethylAid* [25], and probes with a high detection *P* value (> 0.01), probes with a low bead count (< 3 beads) and probes with a low success rate (missing in > 95% of the samples) were set to missing. Subsequently, imputation [26] was performed to impute the missing values. Functional normalization, as implemented in the *minfi* package, was used on a random subset of 1000 samples together with the melanoma samples [27]. A detailed description of the 450K DNA methylation pre-processing steps is available from the https://molepi.github.io/DNAmArray_workflow/. Sample specific aberrant melanoma CpGs were detected using a *t* test for comparing a single melanoma case to the 1000 BIOS controls [28]. In order to control for the number of tests, a very stringent cut-off, 1.03 × 10^−9^ (0.01/(number of probes on array × 2)), was used. After the bioinformatic analysis, a set of 13 hypermethylated CpGs and 164 hypomethylated CpG sites was obtained. The list of significant CpGs was further reduced by only considering significant co-segregating CpGs with an absolute *β* value difference of 0.2 when compared to BIOS controls (Δ*β* value ≥ 0.2 in 2 members of at least one family). To be considered as a putative epimutation, a CpG should meet the following criteria. CpG probes on chromosome X were excluded (as they reflect X-chromosome inactivation in females). Only CpGs in promoter regions (retrieved from Illumina annotation for gene promoters, “promoter_associated in regulatory_feature_group field”) of the genes were selected. Both members of a family were required to harbor the hypo/hypermethylation, since we are looking at high penetrance epigenetic events. If there is a single nucleotide variant (SNV) within a window of 100 bp around the CpG (that can either influence/impair the probe binding or reveal the presence of a genetic variant around the epigenetically altered CpG, that in this case would be the so-called second epimutation), this CpG must be excluded. The SNV data were retrieved from dbSNP Release 153. This resulted in 6 hypermethylated CpGs and 35 hypomethylated CpGs, which were compared with lists of cancer-related genes according to Cancer Gene Census (http://cancer.sanger.ac.uk/census, accessed August 2019) and cancer predisposition genes suggested by Rahman [19]. We have checked whether some CpG sites of interest were part of predicted transcription factor binding motifs using the TFBIND tool [22]. We also aimed at identifying differentially methylated regions. For that, we assessed the probes assigned for promoter regions according to the annotation of 450K array. There were 13,715 genes with probes assigned to promoters. On average, each of these promoters contained 6.7 probes. We averaged all the probes assigned for each gene promoter and compared with the average of the same promoter in BIOS controls.

## Supplementary information

**Additional file 1: Table S1.** Methylation levels (β-value) and cancer genes information of all 6 significant upregulated CpGs in all subjects (*n*=10). **Table S2.** Methylation levels (β-value) and cancer genes information of all 35 significant downregulated CpGs in all subjects (n=10).

**Additional file 2: Figure S1.** Dutch melanoma families included in the whole-genome sequencing analysis. Left quarter red panel: cutaneous malignant melanoma (CMM) only; left quarter yellow panel: multiple melanoma (patient number I_2 and III_1 included in our study, see Table 1); right quarter blue panel: other cancer(s). The melanoma cases subjected to whole-exome sequencing included in this study are indicated by ‘WGS’. Age at CMM diagnosis is given between brackets. **A.** Family I **B.** Family II **C.** Family III **D.** Family IV and **E.** Family V.

**Additional file 3: Figure S2.** Methylation levels (β-value) across the entire sequence of all imprinted genes (http://www.geneimprint.com/site/genes-by-species, accessed August 2019) assessed by 450 K array. In the upper part of each plot, the gene structure is represented in red and promoter region in blue. The light grey arrow represents the transcription direction of the gene. For each CpG, the BIOS values are represented by the black vertical line with upper (average + 1 SD) and lower limits (average – 1SD). The families are represented as a X of different colours (Family I – green, Family II – blue, Family III – yellow, Family IV – light purple, Family V – dark blue). To be considered as significantly different from the BIOS, the families symbols must go beyond the small black horizontal line (average ± 5.65 SD). Genes with more than 10 CpG sites assessed by 450 K array, were represented by 10 randomly selected CpGs.

## Data Availability

The dataset supporting the conclusions of this article, Biobank-based Integrative Omics Studies (BIOS) consortium, is available in the European Genome-phenome Archive (EGA) under accession EGAS00001001077 (https://www.ebi.ac.uk/ega/studies/EGAS00001001077).

## Abbreviations

BIOS Consortium: Biobank-based Integrative Omics Studies
EGA: European Genome-phenome Archive
HNPCC: Hereditary nonpolyposis colorectal cancer
SNV: Single nucleotide variant
