# Supplementary material for: Genome-wide analysis of constitutional DNA methylation in familial melanoma
Source: Clin Epigenetics. 2020 Mar 6;12:43. doi: 10.1186/s13148-020-00831-7 (PMC7060565; doi:10.1186/s13148-020-00831-7)
Supplement: Supplementary file 2 — Additional file 2: Figure S1. Dutch melanoma families included in the whole-genome sequencing analysis. Left quarter red panel: cutaneous malignant melanoma (CMM) only; left quarter yellow panel: multiple melanoma (patient number I_2 and III_1 included in our study, see Table 1); right quarter blue panel: other cancer(s). The melanoma cases subjected to whole-exome sequencing included in this study are indicated by ‘WGS’. Age at CMM diagnosis is given between brackets. A. Family I B. Family II C. Family III D. Family IV and E. Family V. [file 13148_2020_831_MOESM2_ESM.pdf]

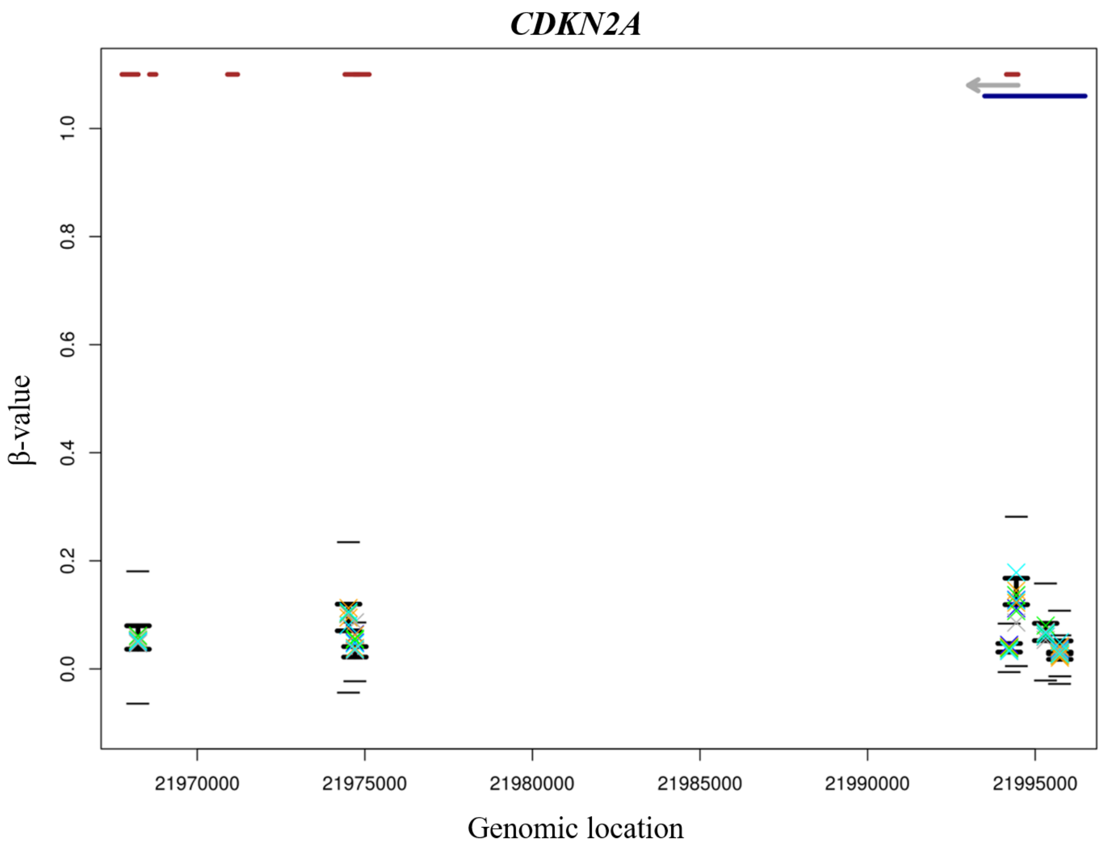
CDKN2A
β-value
0.0
0.2
0.4
0.6
0.8
1.0
21970000
21975000
21980000
21985000
21990000
21995000
Genomic location

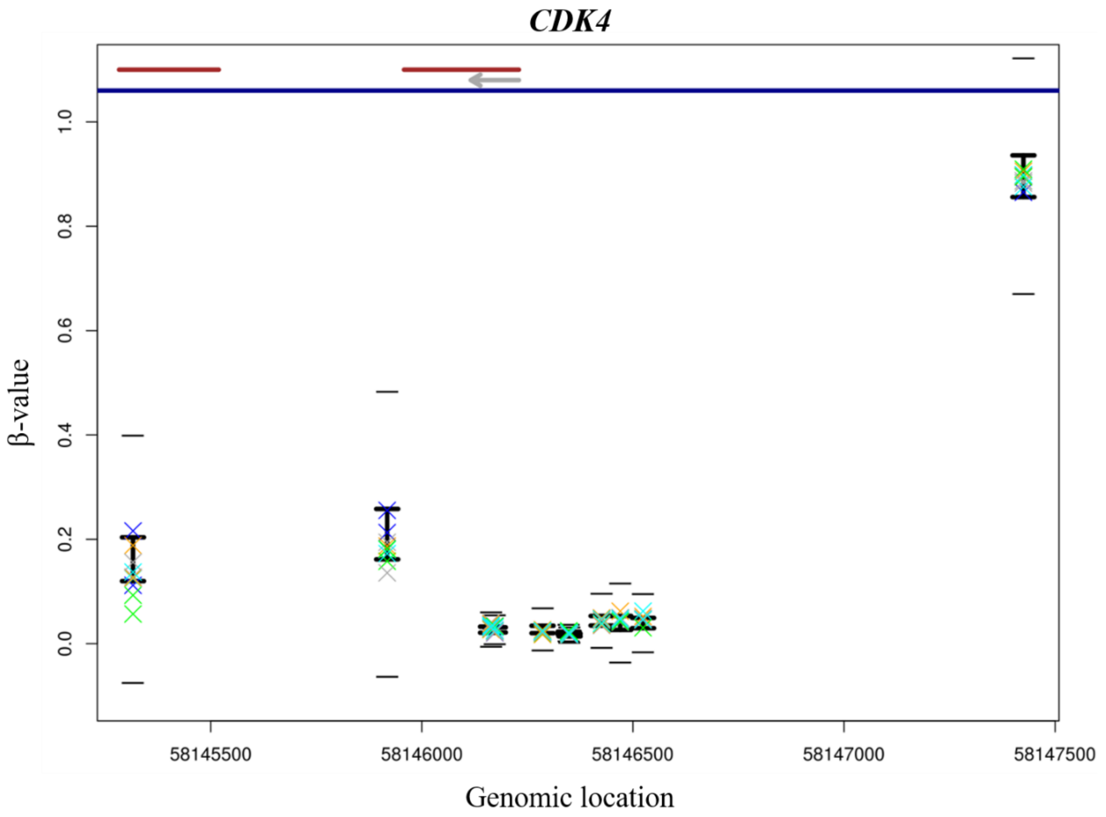
CDK4
β-value
0.0
0.2
0.4
0.6
0.8
1.0
58145500
58146000
58146500
58147000
58147500
Genomic location

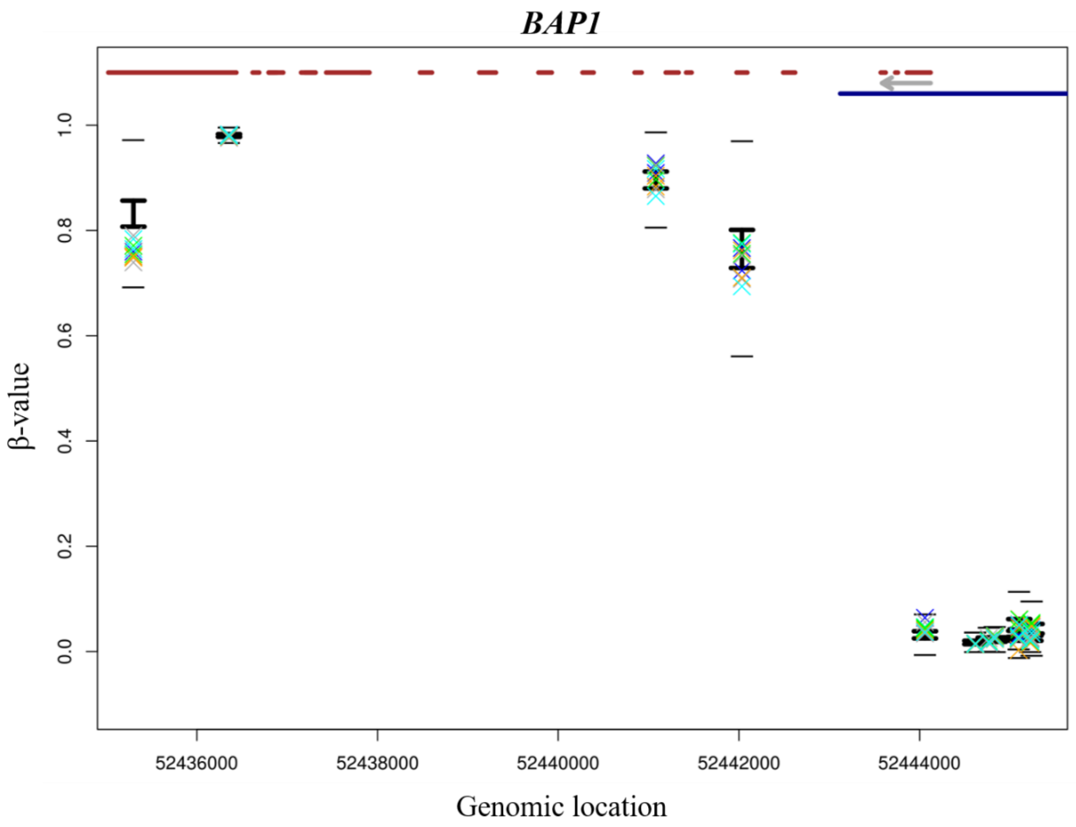
BAP1
β-value
0.0
0.2
0.4
0.6
0.8
1.0
52436000
52438000
52440000
52442000
52444000
Genomic location

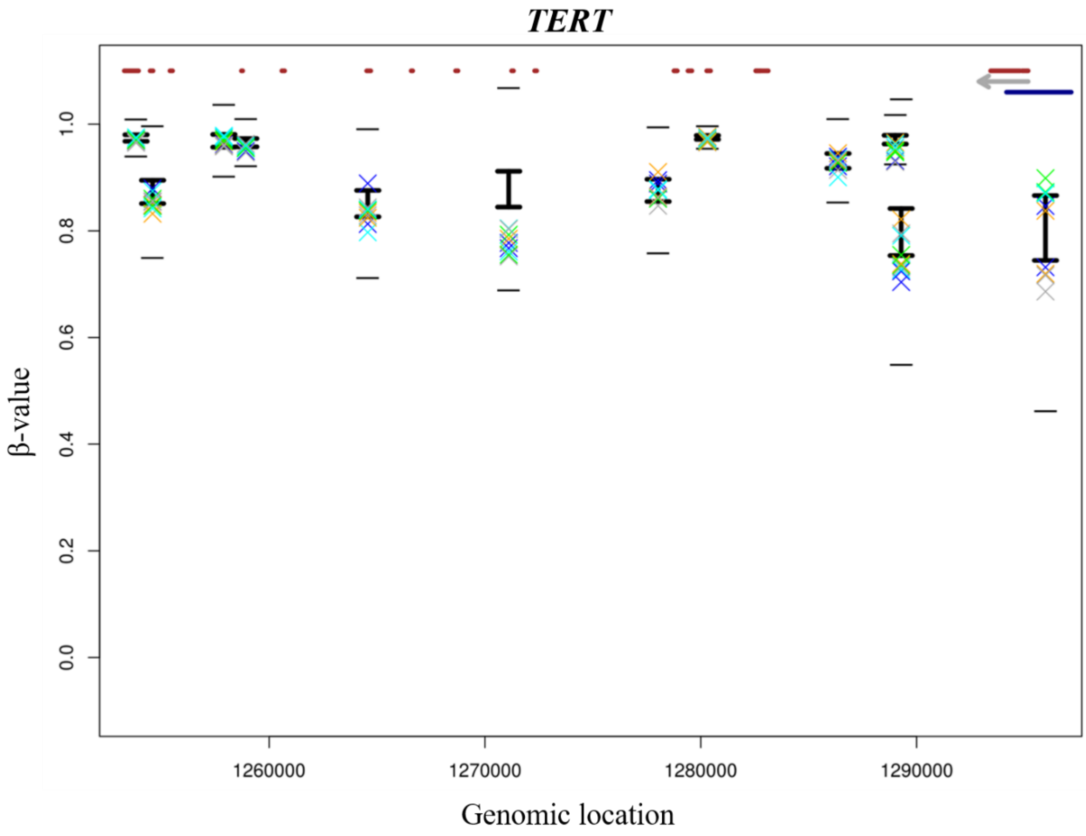
TERT
β-value
0.0
0.2
0.4
0.6
0.8
1.0
1260000
1270000
1280000
1290000
Genomic location

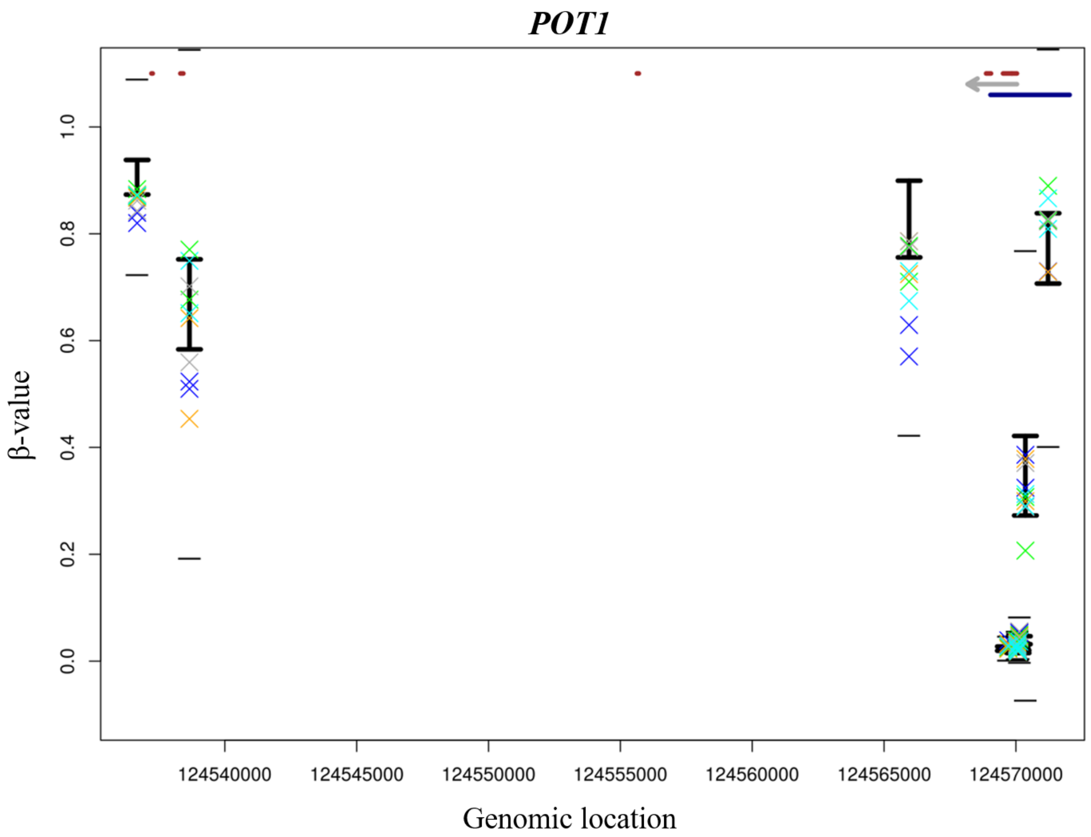
POT1
β-value
0.0
0.2
0.4
0.6
0.8
1.0
124540000
124545000
124550000
124555000
124560000
124565000
124570000
Genomic location

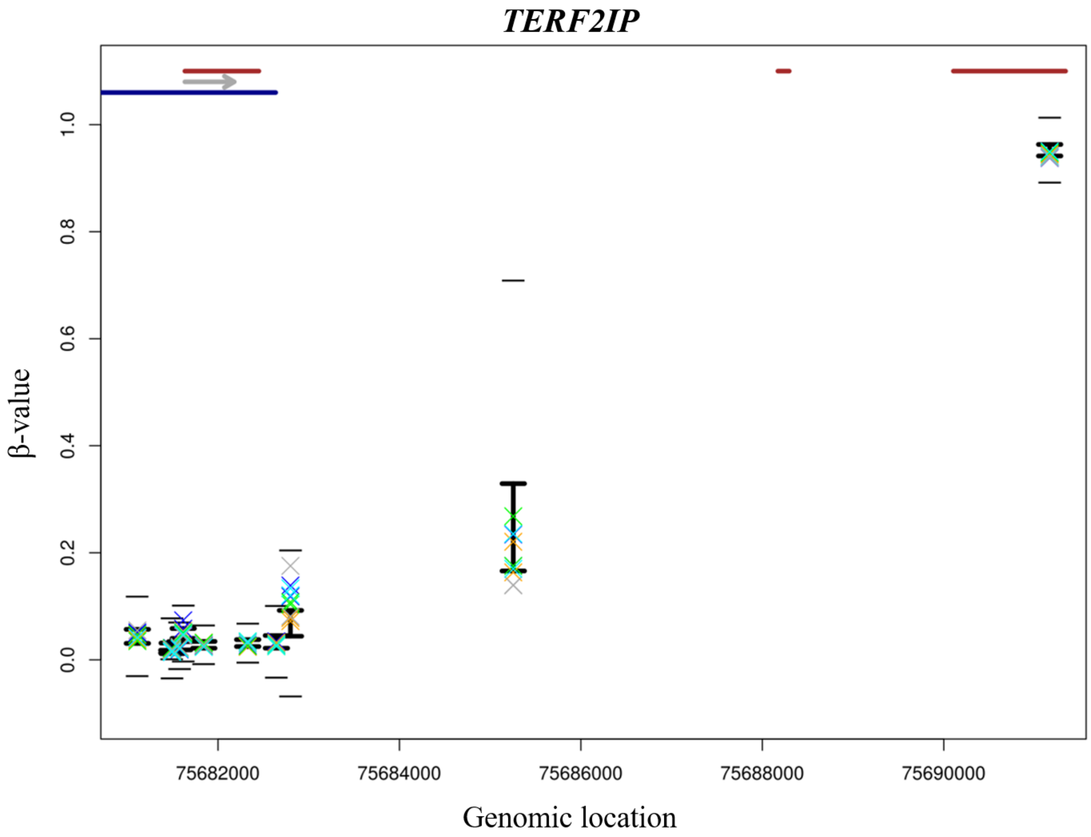
TERF2IP
β-value
0.0
0.2
0.4
0.6
0.8
1.0
75682000
75684000
75686000
75688000
75690000
Genomic location

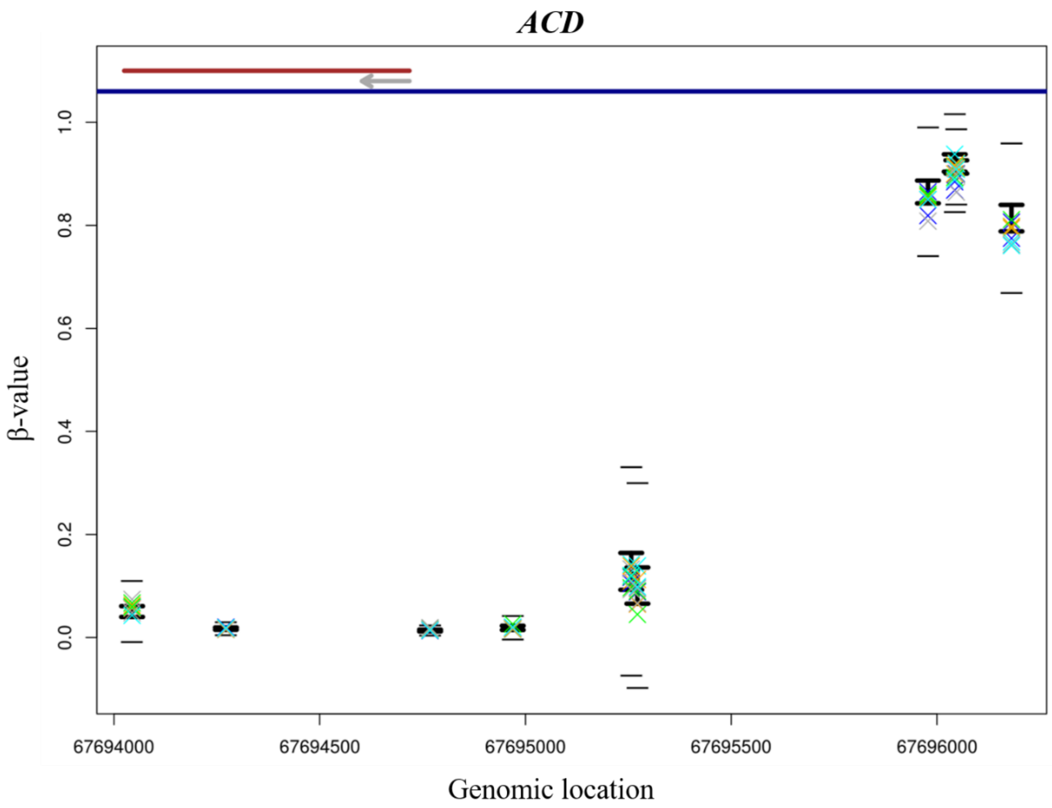

ACD
1.0
0.8
0.6
0.4
0.2
0.0
β-value
67694000
67694500
67695000
67695500
67696000
Genomic location

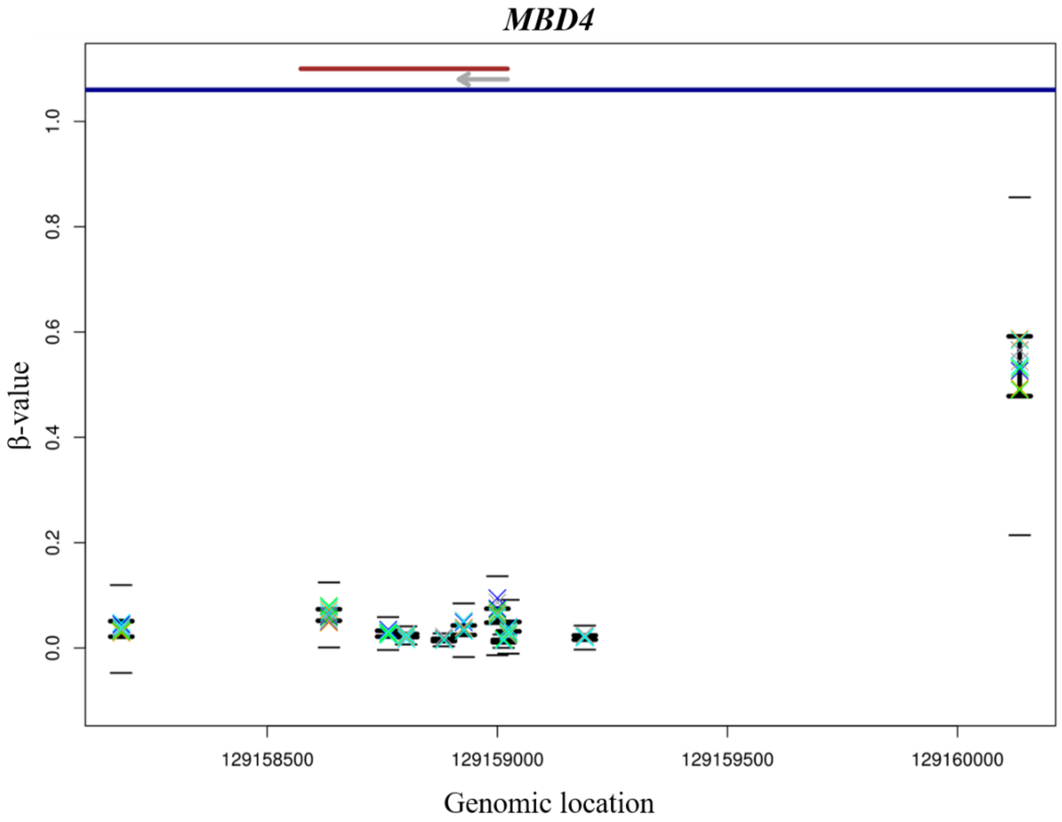

MBD4
1.0
0.8
0.6
0.4
0.2
0.0
β-value
129158500
129159000
129159500
129160000
Genomic location

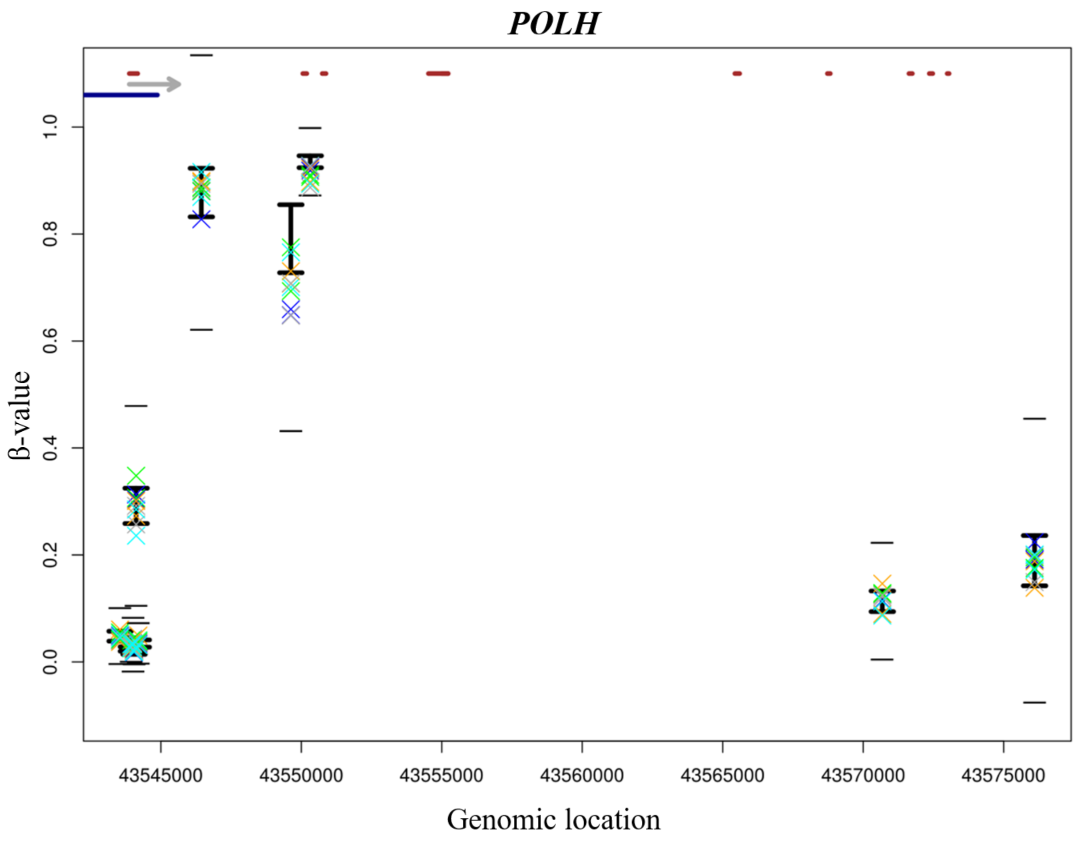
POLH
β-value
1.0
0.8
0.6
0.4
0.2
0.0
43545000
43550000
43555000
43560000
43565000
43570000
43575000
Genomic location

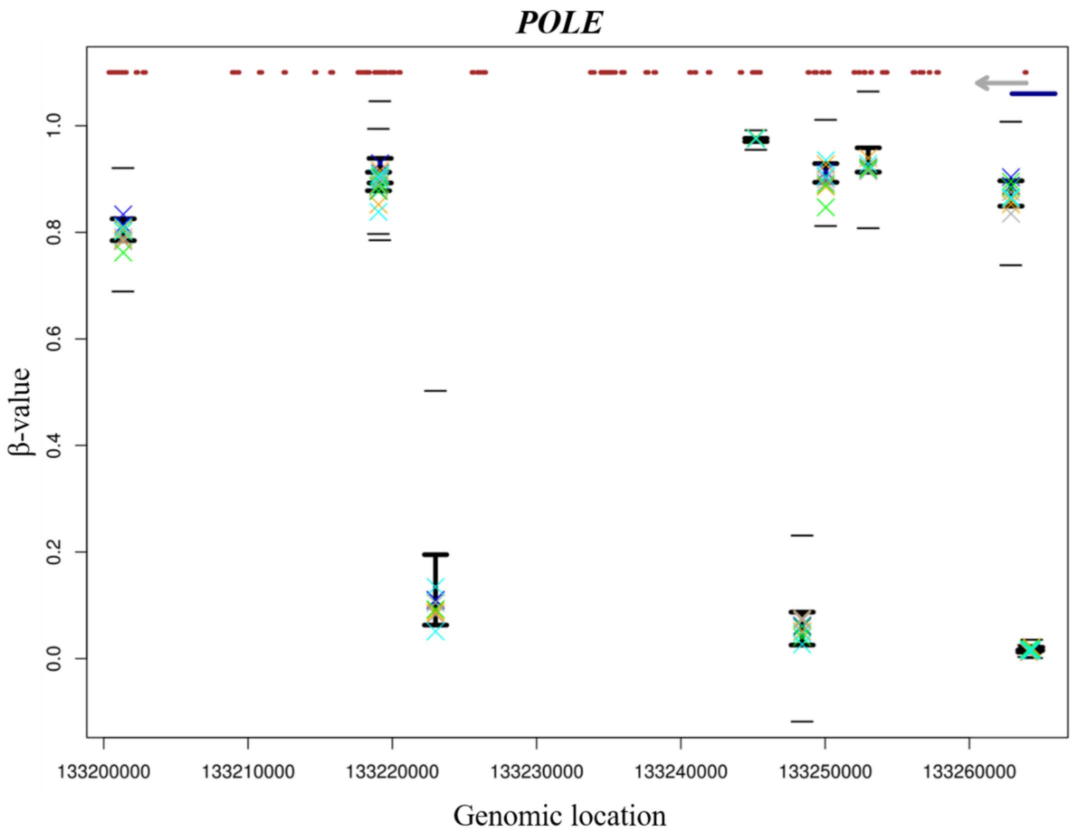
POLE
β-value
1.0
0.8
0.6
0.4
0.2
0.0
133200000
133210000
133220000
133230000
133240000
133250000
133260000
Genomic location

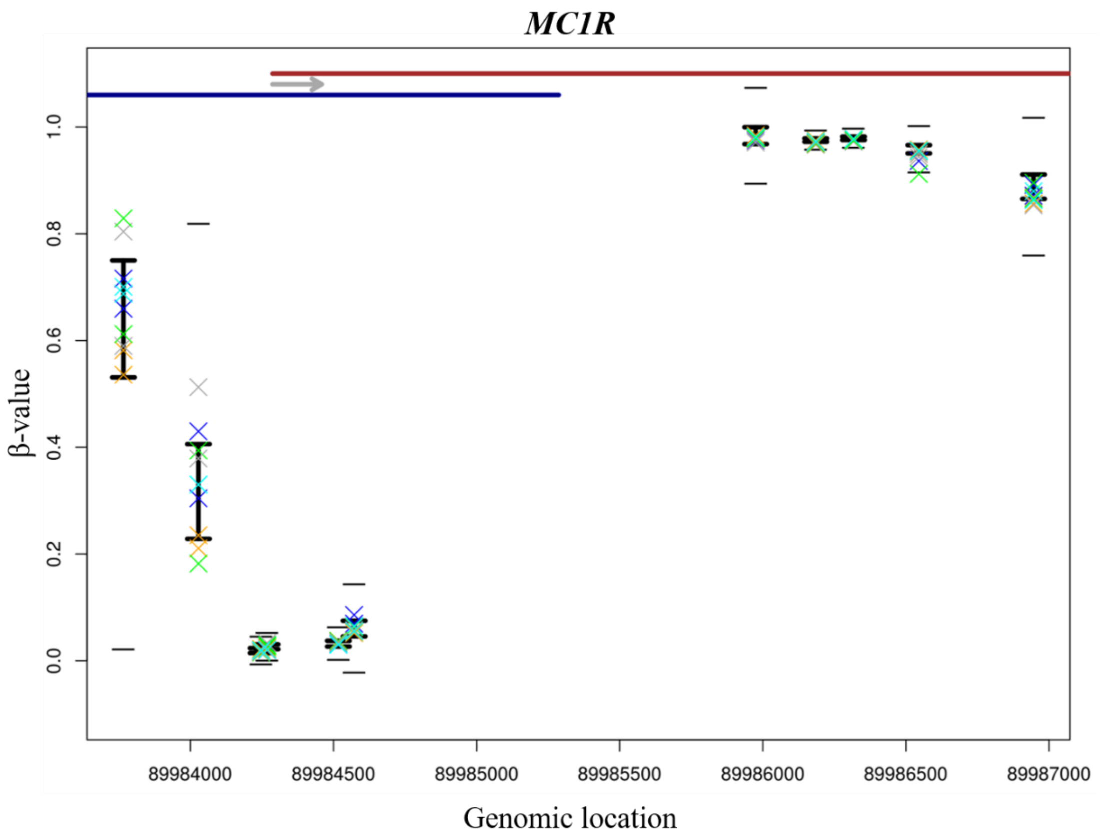
MC1R
β-value
1.0
0.8
0.6
0.4
0.2
0.0
89984000
89984500
89985000
89985500
89986000
89986500
89987000
Genomic location

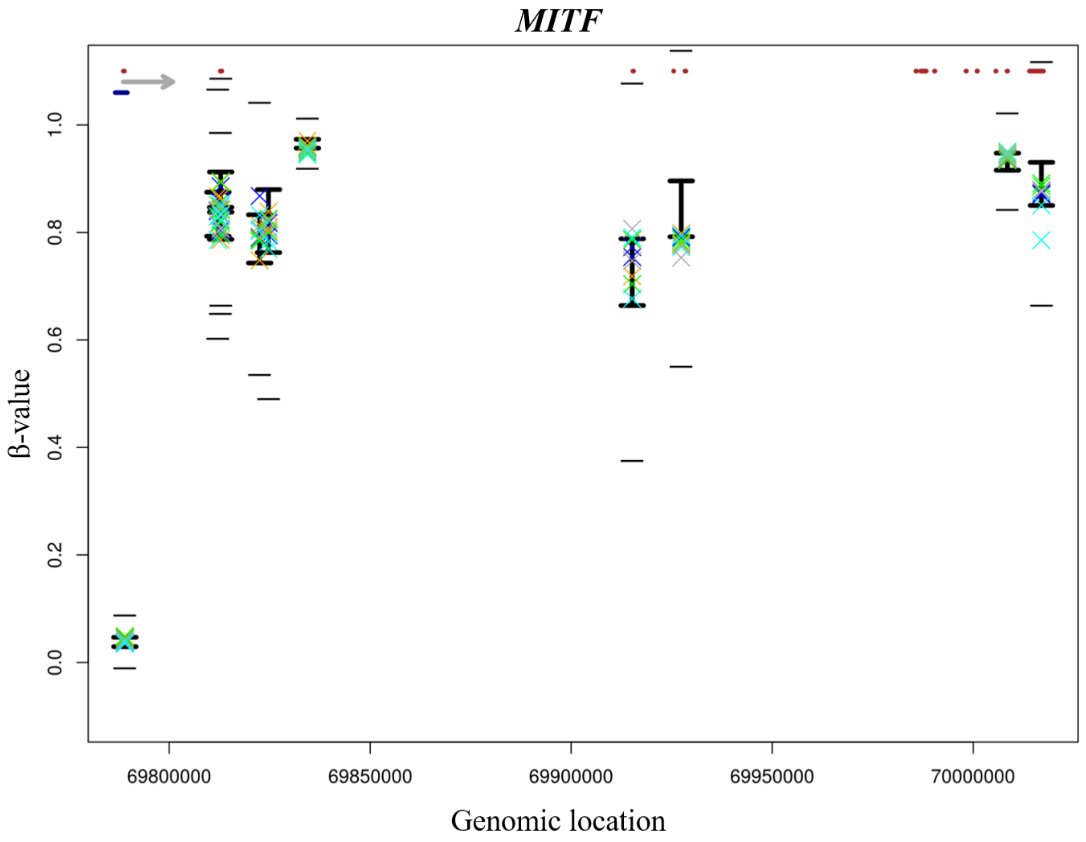
MITF
β-value
1.0
0.8
0.6
0.4
0.2
0.0
69800000
69850000
69900000
69950000
70000000
Genomic location

Extended Figure 1. Methylation levels (β-value) across the entire sequence of all established melanoma predisposition genes. In the upper part of each plot, the gene structure is represented in dark red and promoter region ("Promoter_associated" feature retrieved from Illumina annotation) in blue. The light grey arrow represents the transcription direction of the gene. For each CpG, the BIOS values are represented by the black vertical line with upper (average + 1 SD) and lower limits (average – 1SD). The families are represented as a X of different colours (Family I – green, Family II – blue, Family III – yellow, Family IV – light purple, Family V – dark blue). To be considered as significantly different from the BIOS, the families symbols must go beyond the small black horizontal line (average ± 5.65 SD). Genes with more than 10 CpG sites assessed by 450K array, were represented by 10 randomly selected CpGs.
