# Supplementary material for: Genome-wide analysis of constitutional DNA methylation in familial melanoma
Source: Clin Epigenetics. 2020 Mar 6;12:43. doi: 10.1186/s13148-020-00831-7 (PMC7060565; doi:10.1186/s13148-020-00831-7)
Supplement: Supplementary file 3 — Additional file 3: Figure S2. Methylation levels (β-value) across the entire sequence of all imprinted genes (http://www.geneimprint.com/site/genes-by-species, accessed August 2019) assessed by 450 K array. In the upper part of each plot, the gene structure is represented in red and promoter region in blue. The light grey arrow represents the transcription direction of the gene. For each CpG, the BIOS values are represented by the black vertical line with upper (average + 1 SD) and lower limits (average – 1SD). The families are represented as a X of different colours (Family I – green, Family II – blue, Family III – yellow, Family IV – light purple, Family V – dark blue). To be considered as significantly different from the BIOS, the families symbols must go beyond the small black horizontal line (average ± 5.65 SD). Genes with more than 10 CpG sites assessed by 450 K array, were represented by 10 randomly selected CpGs. [file 13148_2020_831_MOESM3_ESM.pdf]

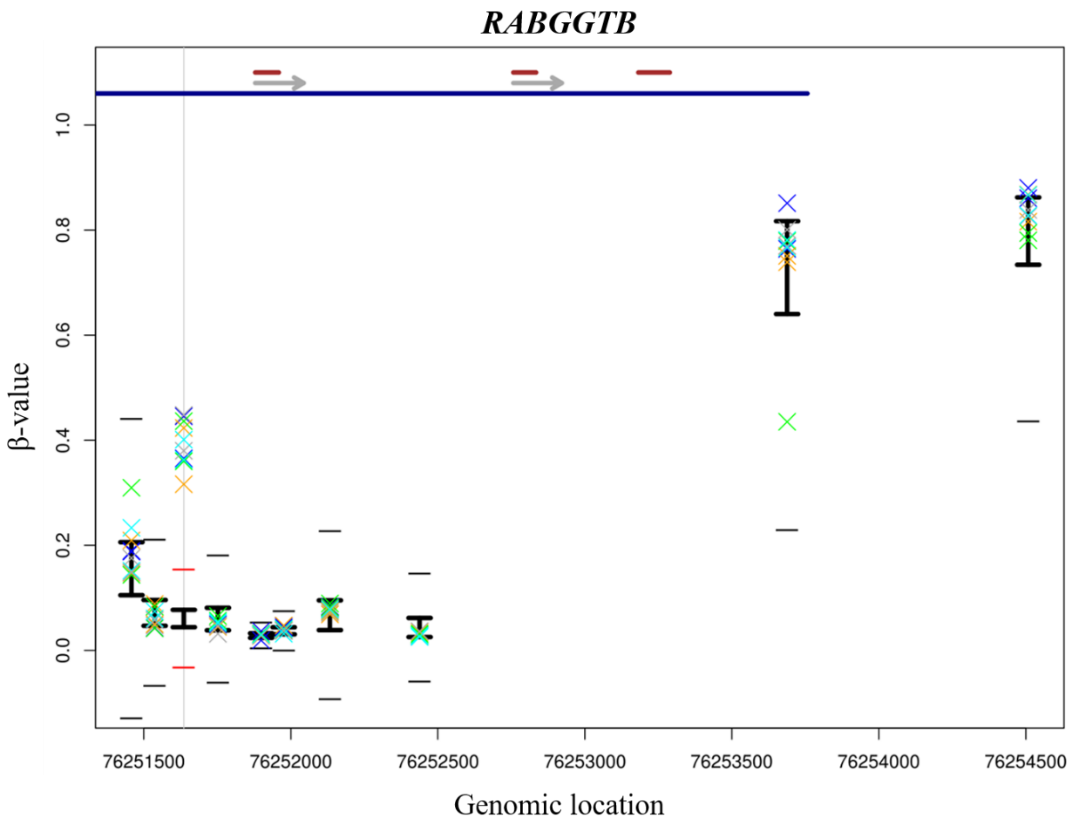
RABGGTB
β-value
1.0
0.8
0.6
0.4
0.2
0.0
76251500
76252000
76252500
76253000
76253500
76254000
76254500
Genomic location

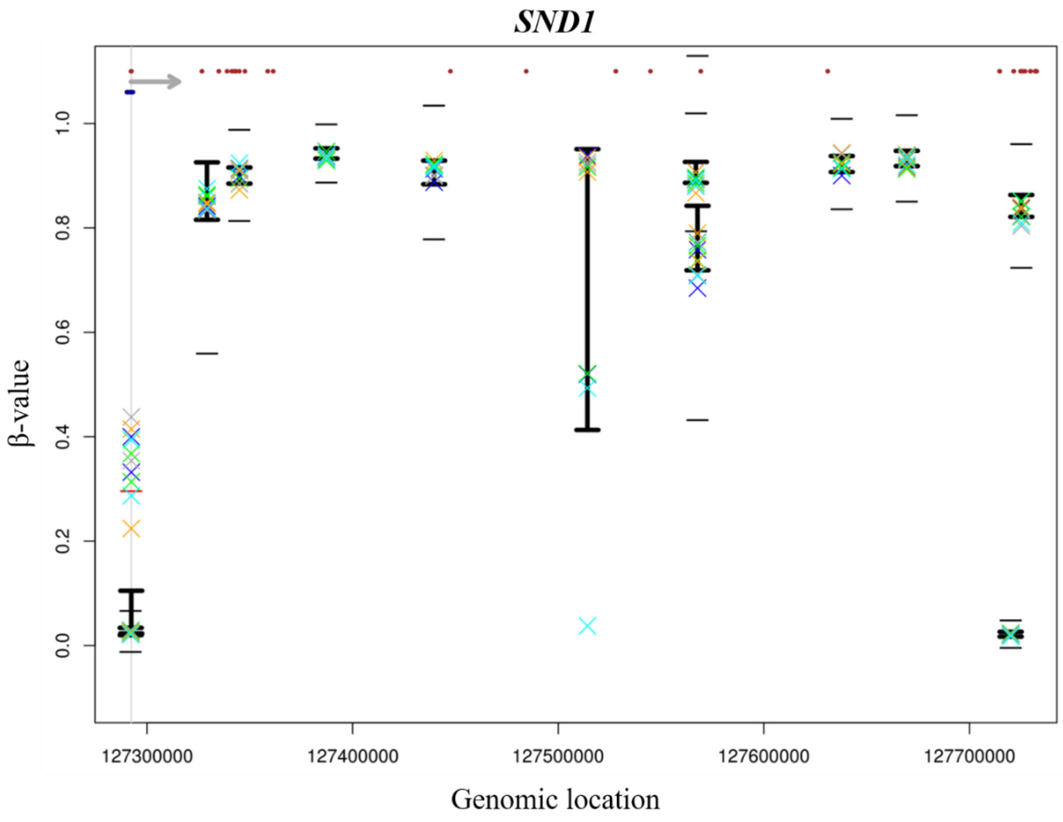
SND1
β-value
1.0
0.8
0.6
0.4
0.2
0.0
127300000
127400000
127500000
127600000
127700000
Genomic location

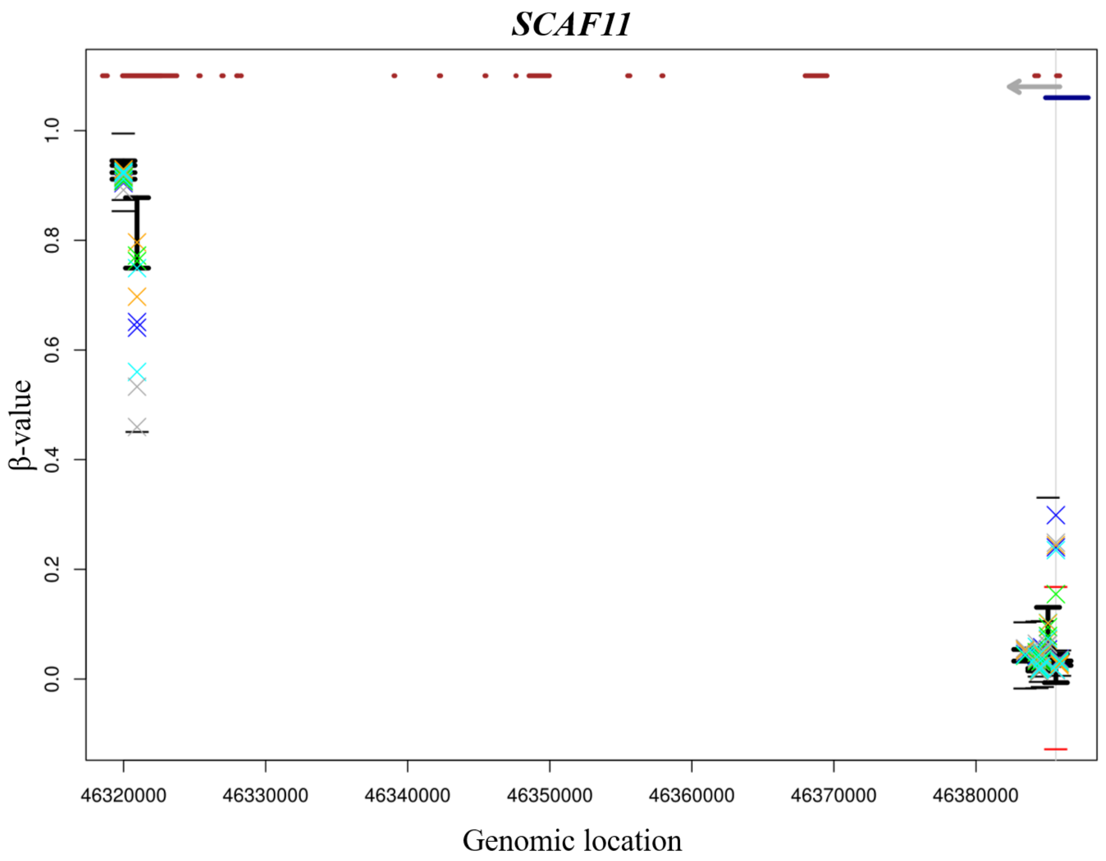
SCAF11
β-value
1.0
0.8
0.6
0.4
0.2
0.0
46320000
46330000
46340000
46350000
46360000
46370000
46380000
Genomic location

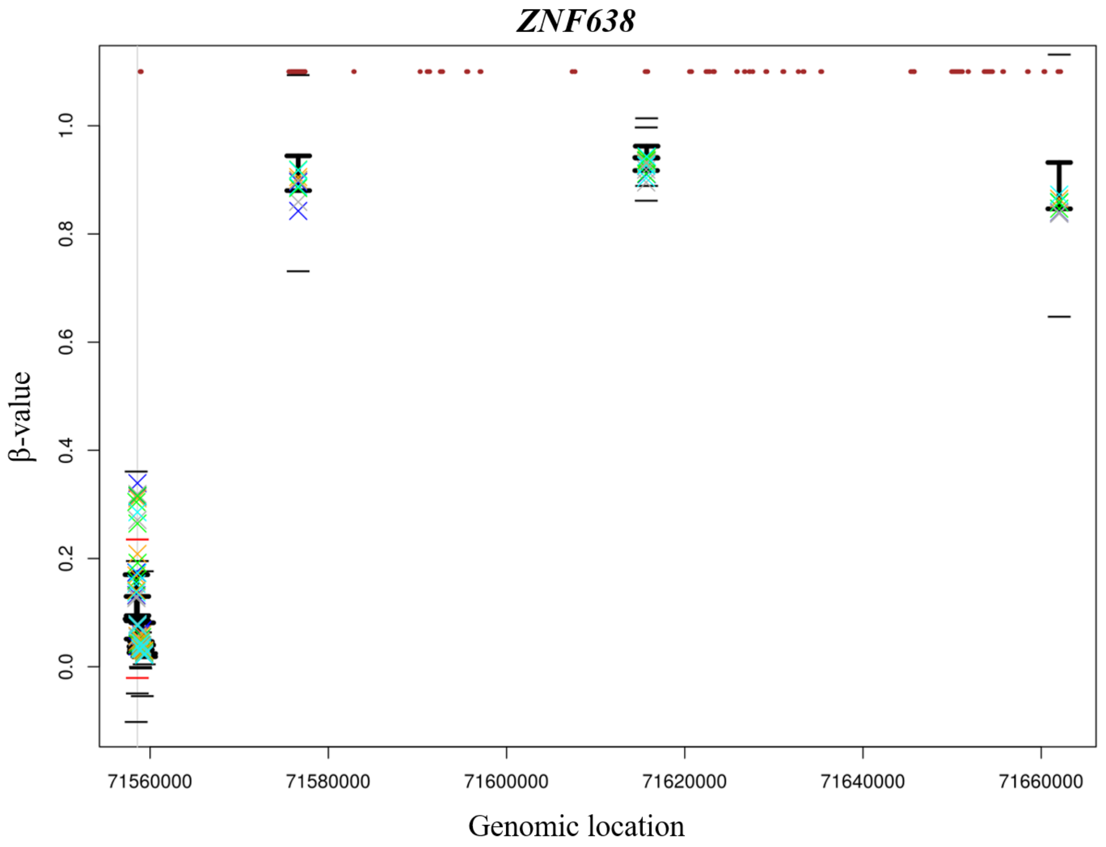
ZNF638
β-value
1.0
0.8
0.6
0.4
0.2
0.0
71560000
71580000
71600000
71620000
71640000
71660000
Genomic location

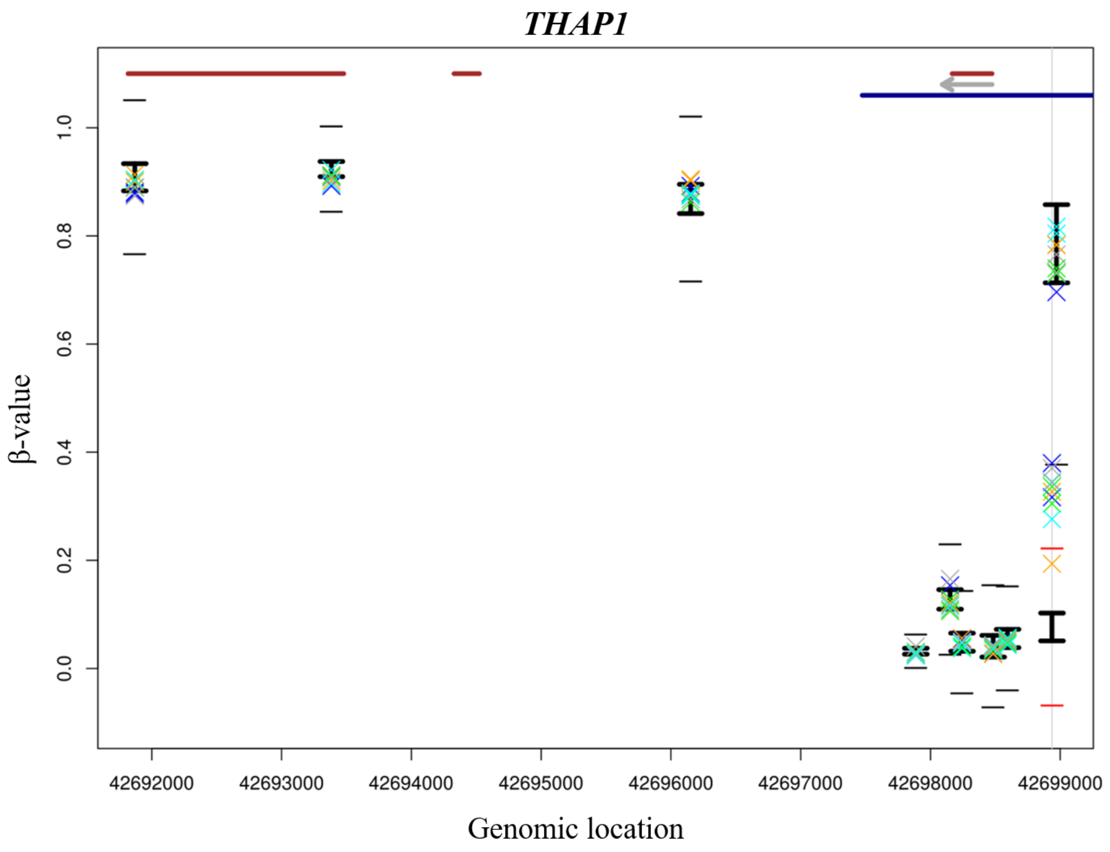
THAP1
β-value
1.0
0.8
0.6
0.4
0.2
0.0
42692000
42693000
42694000
42695000
42696000
42697000
42698000
42699000
Genomic location

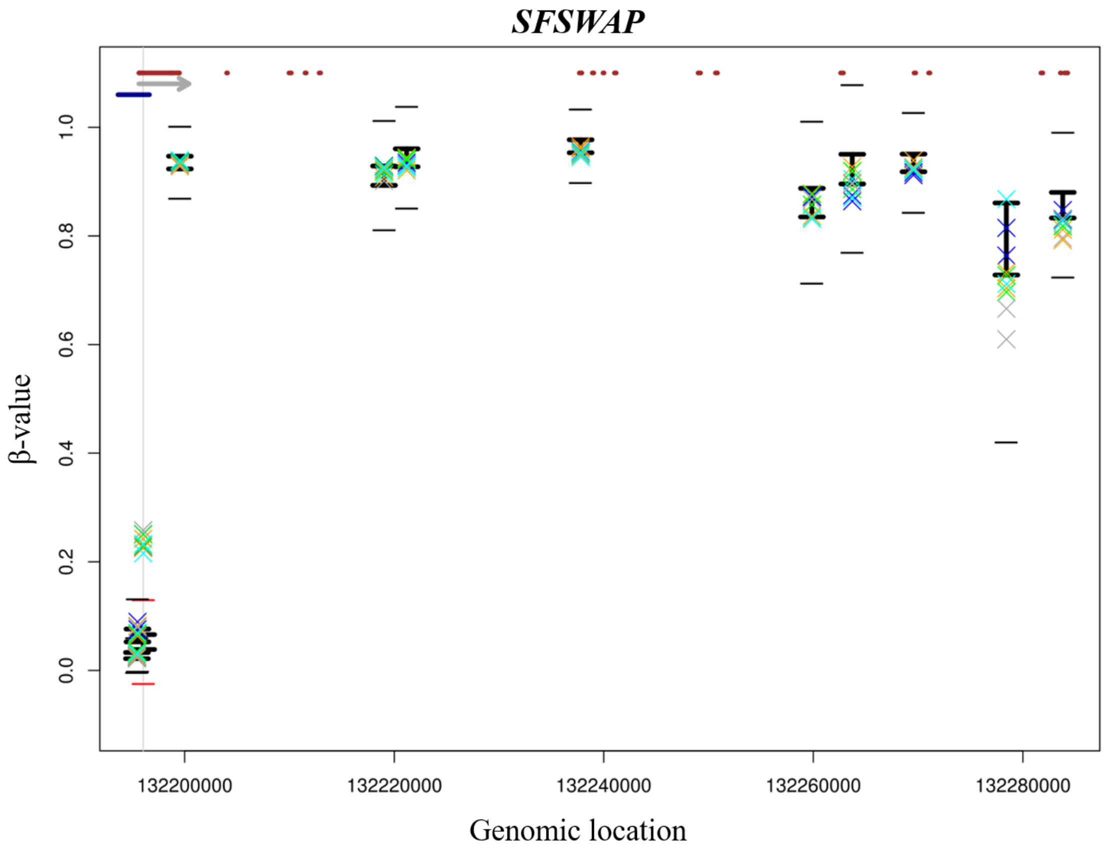
SFSWAP
β-value
1.0
0.8
0.6
0.4
0.2
0.0
132200000
132220000
132240000
132260000
132280000
Genomic location

Extended Figure 2. Methylation levels (β-value) in all 6 significant upregulated CpGs located in the promoter regions of the genes. In the upper part of each plot, the gene structure is represented in dark red and promoter region (“Promoter_associated” feature retrieved from Illumina annotation) in blue. The light grey arrow represents the transcription direction of the gene. For each CpG, the BIOS values are represented by the black vertical line with upper (average + 1 SD) and lower limits (average – 1SD). The families are represented as a X of different colours (Family I – green, Family II – blue, Family III – yellow, Family IV – light purple, Family V – dark blue). To be considered as significantly different from the BIOS, the families symbols must go beyond the small black horizontal line (average ± 5.65 SD). Genes with more than 10 CpG sites assessed by 450K array, were represented by 10 randomly selected CpGs. The upregulated CpG in each plot is aligned with a vertical light grey line and, in this case, the little horizontal lines become red since the families’ symbols exceeded these limits.
